# Artificial Pneumothorax*Continued from January Number.

**Published:** 1915-04

**Authors:** A. G. Shortle


### TEXAS MEDICAL JOURNAL

Dr. F. E. Daniel, Founder. Established July, 1885

MRS. F. E. DANIEL, - Publisher and Managing Editor

Published Monthly.—Subscription, $1.00 a Year.

VOL. XXX AUSTIN, APRIL, 1915 NO. 10

The publisher is not responsible for views of contributors

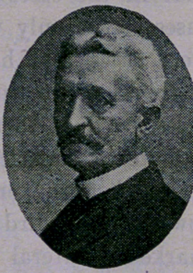

### Original Articles.

### Artificial Pneumothorax.*

BY A. G. SHORTLE, M. D.

Results:

Taking up now the matter that really counts when considering a new treatment, i. e.: the *results,* I can say that in almost three years of its use I have found it the one treatment that produces marked and certain results in the advanced consumptive.

All the recognized treatments for tuberculosis are so slow in their action that the physician can never feel sure if his treatment has been a factor in the improvement of his patient, but with artificial pneumothorax the most marked symptomatic improvement may be produced within a few days. I have seen fever of 103½ reduced to normal within from two to five days. Sputum that has persisted for a year or more will often disappear within two months. Indeed, the early improvement is so remarkable that patients are likely to become over sanguine and be unprepared for some of the complications that may occur later.

About the only unfavorable symptom in the early weeks of the pneumothorax treatment is the matter of weight. It is not uncommon for a patient to have less fever, less cough and improve in every way but lose weight. This is, in part at least, due to a poor appetite caused by displacing the stomach to one side and possibly to some pressure or pulling of the pneumogastric nerve. Later the stomach becomes accustomed to the changed position and the patient will gain weight.

*Continued from January Number.

Coming to the ultimate result, if one will keep in mind the fact that in the cases operated, by me at least, all were unfavorable cases. Not only were they nearly all third stage cases, but they were cases that had failed to improve under all the recognized methods of treatment and most of them had been sanatorium cases. All of them had had the advantage of probably the best climate in America for tuberculosis. The few second stage cases operated were very unfavorable cases that were fast advancing toward the third stage. Eighty-seven per cent of them were marked bi-lateral cases.

I have attempted the use of artificial pneumothorax in 94 cases, but about 20 per cent of these were inoperable on account of extensive adhesions, while a number of others allowed of only such incomplete collapse that the treatment was discontinued after one to three or four fillings, so there remains only 68 cases in which the collapse was considerable enough to warrant the continuance of the treatment. Of the 68 cases treated during the last three years, 18 are dead. Two of these never gave the treatment a fair trial but were persuaded by well meaning friends to abandon it. One of these two, I feel certain, would have been an arrest by this time, as he was making most marked progress under the treatment. Three of those now living quit treatment before it had a fair trial and I do not know their condition; four were made worse by the treatment; in seven we abandoned treatment as not proving of value, though apparently the patients were no worse. Eight are symptom free and working. Of those still under treatment, nine are symptom free and the treatment is being continued only to insure permanency; five are not symptom free but much improved in every way, and fourteen are still under treatment, but it is too early to predict results.

Complications:

Of the unfavorable complications ensuing during the course of treatment the most common is that of pleurisy with effusion. This occurs in a large per cent of cases, certainly 50 per cent or more of those treated a year.

This is probably largely due to tubercle forming on the pleural surface of the lung and in the uncollapsed lung it would probably become attached to the opposite pleura and so the spread of the tubercle would be limited, but in the collapsed case preventive action cannot take place.

The introduction of cold nitrogen, the frequent punctures of the pleura in a limited space, and a number of other reasons are given for its occurrence.

However, the first symptoms of fever, pain and oppression are more alarming than the results are serious. Most of the cases only required to be drained a few times and the lung compression continued and the trouble was soon over.

A more dangerous complication is the occurrence of spontaneous pneumothorax during the course of the artificial one. This is possibly caused by the pulling of adhesive bands tearing the lung, though in our experience it has occurred not in cases having a high positive pressure, as one would expect, but in those having a negative or neutral pressure. This may cause no special trouble but is likely to end in pus infection of the pleura which is to be treated as would any other pleural empyema.

IN CONCLUSION.

After almost three years with this method I feel that for a certain percentage of the advanced consumptives it offers the only hope of improvement, and it is far ahead of any other means we have for combating pulmonary tuberculosis in the suitable case. Later it is possible that the early case will be treated by this method as well as the late, and I think it will, but for the present it is more conservative to use it only in the unfavorable advanced case.

